# Taking Sleeping Pills and the Risk of Chronic Kidney Disease: A Nationwide Population-Based Retrospective Cohort Study

**DOI:** 10.3389/fphar.2020.524113

**Published:** 2021-01-25

**Authors:** Chen-Yi Liao, Chi-Hsiang Chung, Kuo-Cheng Lu, Cheng-Yi Cheng, Sung-Sen Yang, Wu-Chien Chien, Chia-Chao Wu

**Affiliations:** ^1^ Division of Nephrology, Department of Internal Medicine, Kaohsiung Armed Forces General Hospital, Kaohsiung, Taiwan; ^2^ Division of Nephrology, Department of Internal Medicine, Tri-Service General Hospital, National Defense Medical Center, Taipei, Taiwan; ^3^ School of Public Health, National Defense Medical Center, Taipei, Taiwan; ^4^ Taiwanese Injury Prevention and Safety Promotion Association, Taipei, Taiwan; ^5^ Division of Nephrology, Department of Medicine, Taipei Tzu Chi Hospital, Buddhist Tzu Chi Medical Foundation, New Taipei, Taiwan; ^6^ PET Center and Department of Nuclear Medicine, Tri-Service General Hospital, Taipei, Taiwan; ^7^ School of Medicine, National Defense Medical Center, Taipei, Taiwan; ^8^ Department of Medical Research, Tri-Service General Hospital, Taipei, Taiwan; ^9^ Graduate Institute of Medical Sciences, National Defense Medical Center, Taipei, Taiwan; ^10^ Department of Medical Research, Tri-Service General Hospital, National Defense Medical Center, Taipei, Taiwan

**Keywords:** peritoneal dialysis, hemodialysis, end-stage renal disease, chronic kidney disease, sleeping pills

## Abstract

**Background:** Sleeping disorder has been associated with chronic kidney disease (CKD); however, the correlation between sleeping pills use and CKD has not been investigated in-depth yet. This study elucidated the potential association of sleeping pill use with the risk of CKD and CKD progression to end-stage renal disease (ESRD) requiring dialysis.

**Methods:** This study was based on a population-based cohort that included 209,755 sleeping pill users among 989,753 individuals. After applying the exclusion criteria, 186,654 sleeping pill users and 373,308 nonusers were enrolled to monitor the occurrence of CKD. Using a cumulative daily dose, we analyzed the types of sleeping pills related to the risk of CKD and ESRD. Propensity score matching and analysis using Cox proportional hazards regression were performed with adjustments for sex, age, and comorbidities.

**Results:** Sleeping pill use was related to increased CKD risk after adjusting for underlying comorbidities (adjusted hazard ratio [aHR] = 1.806, 95% confidence interval [CI]: 1.617–2.105, *p* < 0.001). With the exception of hyperlipidemia, most comorbidities correlated with an increased risk of CKD. Persistent use of sleeping pills after CKD diagnosis increased the risk of concurrent ESRD (aHR = 7.542; 95% CI: 4.267–10.156; *p* < 0.001). After the subgroup analysis for sleeping pill use, brotizolam (*p* = 0.046), chlordiazepoxide (*p* < 0.001), clonazepam (*p* < 0.001), diazepam (*p* < 0.001), dormicum (*p* < 0.001), estazolam (*p* < 0.001), fludiazepam (*p* < 0.001), flunitrazepam (*p* < 0.001), nitrazepam (*p* < 0.001), trazodone (*p* < 0.001), zolpidem (*p* < 0.001), and zopiclone (*p* < 0.001) were found to have significant correlation with increased CKD risk.

**Conclusion:** Sleeping pill use was related to an increased risk of CKD and ESRD. Further studies are necessary to corroborate these findings.

## Introduction

Chronic kidney disease (CKD) is characterized by abnormalities of kidney function or structure presenting for >3 months and associated with health problems depending on the causes, glomerular filtration rate category, and albuminuria category. CKD is known to contribute to the risk of cardiovascular events, cardiovascular mortality, and all-cause mortality. Many drugs have been associated with CKD occurrence. The complex and dynamic relationship between sleeping pills and CKD remains relatively poorly understood. Furthermore, the burdens of sleeping pills on CKD and progression to end-stage renal disease (ESRD) are rarely discussed (Turek et al., 2012). Taiwan is well known for its high prevalence of CKD (11.9%) and ESRD among adults, with a higher prevalence among the elderly (37.2%), which makes them important study subjects. According to the report, the prevalence of sleep disturbances is estimated to be 80% among patients with CKD; thus, the use of sleeping pills may have a significant impact on this group of people (Turek et al., 2012).

Sleeping pills, primarily benzodiazepines, nonbenzodiazepine omega-receptor agonists (zolpidem), tricyclic antidepressants, selective gamma-aminobutyric acid (GABA) agents, and antihistamines, have been widely introduced in an era where sleeping and anxiety disorders are common. Approximately 2–5% of sleeping pill users are young adults, while most are older adults and women. Sleeping pills are well known for their undesired side-effects, including daytime sedation, confusion, cognitive deficits, dependency, withdrawal, rebound symptoms, ataxia, dysarthria, diplopia, and vertigo (Wang et al., 2001). Benzodiazepine can cause various adverse events such as falls, major fractures, and motor vehicle accidents. The elderly are at a particular risk of such adverse events because of poor elimination of the metabolized drugs (Tamblyn et al., 2005). Furthermore, the use of benzodiazepine and zolpidem was associated with a 15% increase in mortality rate. Winkelmayer et al. previously concluded that frequent use of benzodiazepines and zolpidem may lead to higher mortality rates in patients undergoing dialysis (Winkelmayer et al., 2007). Various studies have reported sleep disorders in patients with CKD and those receiving dialysis (Winkelmayer et al., 2007; Natale et al., 2019). Sleep disorders may increase the use of sleeping pills. In patients with CKD and ESRD, the elimination of these drugs may be impaired or shortened, thus leading to a higher risk of adverse events. As the relationship between the cumulative use of sleeping pills and the subsequent risk of CKD and ESRD is still poorly understood, the purpose of this study was to assess the impact of sleeping pill exposure on CKD and further ESRD.

## Materials and Methods

### Data Source

The present study was designed as a longitudinal data study and time-to-event analysis and was conducted over a 13-year period, using data acquired from the National Health Insurance Research Database (NHIRD), which is maintained by the National Health Research Institutes. The NHIRD consists of broad population-based datasets covering 99.9% of the Taiwanese inhabitants. We applied inpatient and outpatient datasets that had diagnoses clarified using the International Classification of Disease, Ninth Revision, Clinical Modification (ICD-9-CM) codes. All data were anonymized to ensure the privacy of all the participants.

### Inclusion and Exclusion Criteria

**FIGURE 1:**
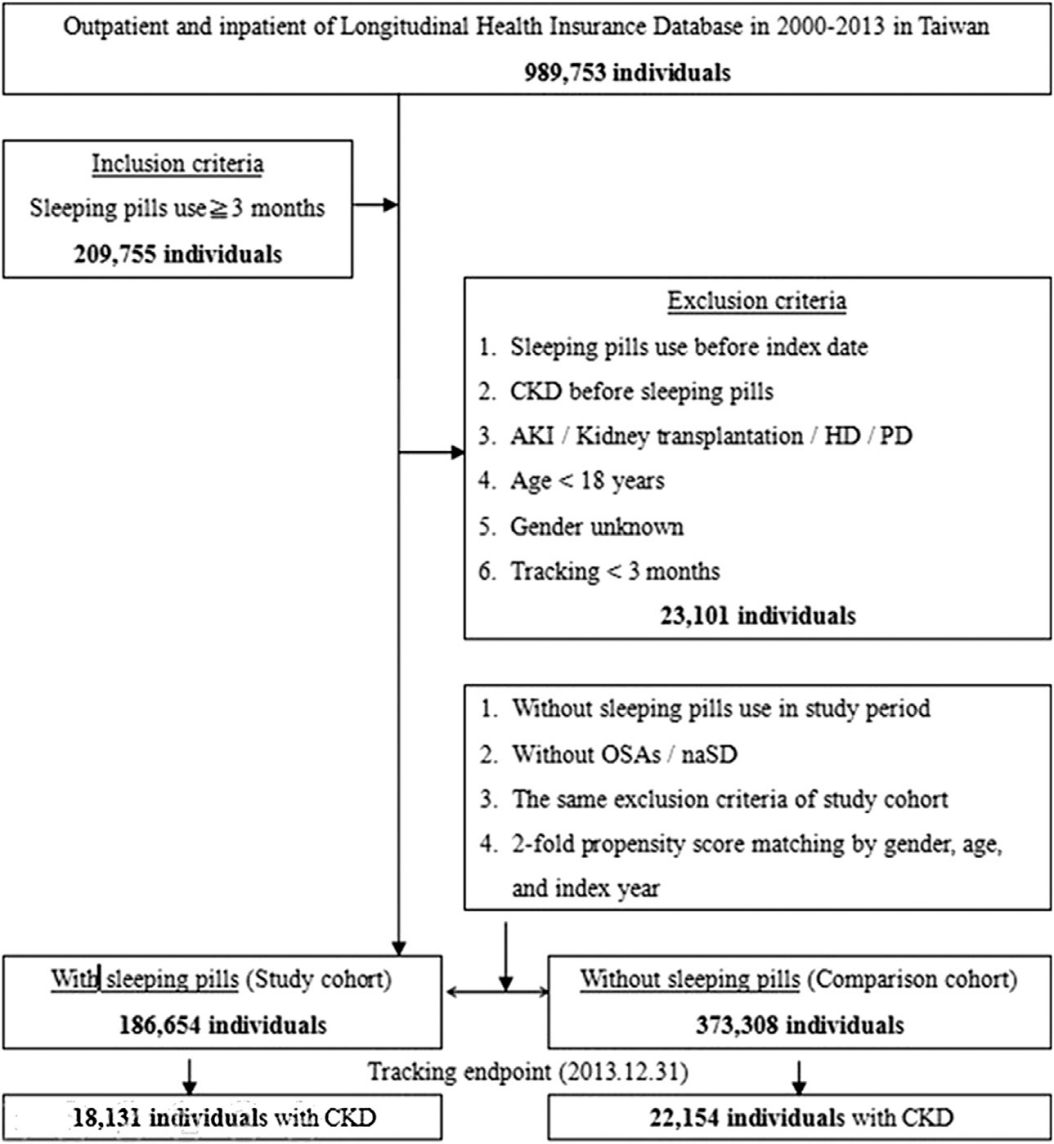
The flowchart of study sample selection from the National Health Insurance Research Database in Taiwan.

The details of the inclusion and exclusion criteria are shown in Figure 1. Those patients who were using sleeping pills were matched with those not using sleeping pills (control group) by propensity score matching (two controls for each sleeping pill user) according to age, sex, and index date, using the same exclusion criteria. The index dates of sleeping pill users and sleeping pill nonusers were matched in the same month. CKD was represented with ICD-9-DM code 585. The patients with ESRD requiring dialysis (ESRDd) were represented with the ICD-9-DM codes for peritoneal dialysis and hemodialysis. With the criteria of absence of evidence of CKD and ESRDd over the previous 3 years (1997–1999), the study subjects were enrolled from January 1, 2000, until December 31, 2013, and defined as newly diagnosed with CKD/ESRDd (Figure 1).

### Outcome and Comorbidities (Potential Confounders)

With the exception of individuals who had withdrawn from the insurance program or were lost to follow-up until the end of 2013, the person-years of follow-up for the patients newly diagnosed with CKD/ESRDd were calculated. Between the index date and January 1, 2013, CKD risk factors were identified as potential confounders, including comorbidities such as diabetes mellitus (ICD-9-CM code 250), hypertension (codes 401–405), chronic liver disease and cirrhosis (LC) (code 571), chronic obstructive pulmonary disease and allied conditions (COPDa; codes 490–496), hyperlipidemia (code 272), congestive heart failure (CHF; code 428), coronary artery disease (CAD; codes 410–414), and stroke (codes 430–438). Nephrotoxic drugs such as NSAIDs (nonsteroidal anti-inflammatory drugs), antibacterial agents (such as aminoglycosides), antifungal or antiviral agents, immunosuppressants, and ACEI (angiotensin-converting enzyme inhibitors)/ARB (angiotensin II receptor blocker) were identified as potential confounders.

### Evaluation of Sleeping Pill Exposure

Those who were not exposed to sleeping pills for >3 years prior to the study period (1997–1999) were included as nonusers of sleeping pills. Regular sleeping pill use was defined as constant sleeping pill use for >90 days after the index date. Sleeping pill use periods with intervals between successive prescriptions exceeding 90 days were abandoned. To judge the association between sleeping pill exposure and further risk of ESRDd, we analyzed the incidence of ESRDd after CKD in those using sleeping pills compared with those not using sleeping pills.

### Statistical Analysis

The incidence rate of CKD (per 10^5^ person-years) was calculated for both cohorts. The chi-square test was used for categorical variables and *t*-test for continuous variables in our study. Multivariate-adjusted Cox proportional hazard regression models were used to analyze associations between sleeping pill use and CKD incidence. The models were adjusted for age, sex, hypertension, diabetes mellitus, stroke, LC, COPDa, CHF, CAD, hyperlipidemia, Charlson comorbidity index (CCI) score, city location, urbanization, level of care, and season. A large number of confounding variables with *p* values of <0.05 after 13 years of follow-up were included in the adjusted model in Table 1. The Kaplan–Meier analysis was applied to assess the cumulative incidence curves of CKD for both cohorts. The log-rank test was applied to assess the differences between the two cohorts. Results are presented as hazard ratios (HR) with 95% confidence intervals (CIs). The SAS statistical software (Version 9.3 for Windows; (SAS Institute, Inc., Cary, NC, United States) was used for all the statistical analyses.

**TABLE 1:** Factors of CKD at the end of the follow-up period stratified by Cox regression.

Variables	With sleeping pills			Without sleeping pills (reference)			Ratio	Adjusted HR (95%CI)	*p* value
Stratified	Events	PYs	Rate	Events	PYs	Rate			
Overall	18,131	1,852,718.01	978.62	22,154	3,841,800,12	576.66	1.697	1.806 (1.617–2.105)	<0.001*
Gender									
Male	10,201	1,033,370.53	987.16	10,865	2,199,862.43	493.89	1.999	2.127 (1.904–2.479)	<0.001*
Female	7,930	819,347.49	967.84	11,289	1,641,937.69	687.54	1.408	1.498 (1.341–1.746)	<0.001*
Age, year									
18–29	301	170,506.59	176.53	128	113,031.10	113.24	1.559	1.659 (1.485–1.934)	<0.001*
30–39	664	238,047.58	278.94	571	383,830.78	148.76	1.875	1.993 (1.784–2.326)	<0.001*
40–49	1,398	295,634.89	472.88	1,099	433,670.36	253.43	1.866	1.985 (1.779–2.139)	<0.001*
50–59	1,736	330,859.05	524.69	1,635	604,582.89	270.43	1.940	2.065 (1.843–2.404)	<0.001*
≧60	14,032	817,669.90	1,716.10	18,721	2,306,684.89	811.60	2.114	2.224 (2.001–2.638)	<0.001*
Comorbidity									
HTN	3,612	38,631,915	934.98	3,598	841,576.62	427.53	2.187	2.327 (2.084–2.715)	<0.001*
DM	5,651	315,775.954	1,789.56	6,200	696,874.46	889.69	2.011	2.143 (1.918–2.499)	<0.001*
CHF	1,586	77,023.53	2,059.11	1,567	179,608.20	872.45	2.360	2.511 (2.240–2.938)	<0.001*
Stroke	1,533	144,467.46	1,061.14	1,786	326,806.34	546.50	1.942	2.066 (1.835–2.421)	<0.001*
COPDa	2,341	175,313.84	1,335.32	1,913	303,463.12	630.39	2.118	2.254 (2.011–2.623)	<0.001*
LC	1,835	104,988.14	1,747.82	1,667	209,419.66	796.01	2.196	2.337 (2.095–2.731)	<0.001*
CAD	2,582	153,669.22	1,680.23	2,612	361,016.51	723.51	2.322	2.474 (2.217–2.890)	<0.001*
Hyperlipidemia	297	58,928.35	504.00	633	123,502.54	512.54	0.983	1.046 (0.937–1.222)	0.108
Without hyperlipidemia	17,834	1,793,789.66	994.21	21,521	3,718,297.58	578.79	1.718	1.828 (1.637–2.131)	<0.001*
Insured premium									
<18,000	16,121	1,820,315.56	885.62	16,252	3,773,394.06	430.70	2.056	2.188 (1.859–2.651)	<0.001*
18,000–34,999	567	26,915.50	2,106.59	1,339	56,153.61	2,384.53	0.883	1.340 (1.010–1.596)	0.039*
≧35,000	1,443	5,486.96	26,298.71	4,563	12,252.45	37,241.53	0.706	0.852 (0.673–1.207)	0.246
Medications									
NSAIDs	6,756	438,592.56	1,540.38	8,382	676,197.76	1,239.58	1.243	1.322 (1.182–1.569)	<0.001*
ACEI and ARB	3,230	231,055.30	1,397.93	4,052	342,282.57	1,183.82	1.181	1.257 (1.126–1.463)	<0.001*
Antibacterial agents	573	45,644.48	1,255.35	897	81,151.43	1,105.34	1.136	1.209 (1.083–1.409)	<0.001*
Antifungal agents	444	33,318.88	1,332.58	765	62,036.12	1,233.15	1.081	1.150 (1.028–1.347)	0.018*
Antiviral agents	599	60,266.22	993.92	1,125	128,154.63	877.85	1.132	1.205 (1.079–1.401)	<0.001*
Immunosuppressive agents	402	30,157.96	1,332.98	711	56,738.13	1,253.13	1.064	1.132 (1.015–1.322)	0.036*

PYs, person-years; rate, per 10^5^ PYs; ratio = rate in cases ÷ rate in controls; adjusted HR, adjusted for all the variables of age, gender, and comorbidities, including the following: HTN, hypertension; DM, diabetes; CHF, congestive heart failure; stroke, COPDa, chronic obstructive pulmonary disease and allied conditions; LC, chronic liver disease and cirrhosis; CAD, coronary artery disease; hyperlipidemia. *p value <0.05.

### Study Approval

This study was approved by the Institutional Review Board of the Tri-Service General Hospital (study approval code: TSGHIRB No. 2-106-05-029). The NHIRD encrypted the personal information of the patients and provided researchers with anonymous identification numbers associated with relevant claim information and prescriptions, medical services received, sex, and date of birth; therefore, patient consent was not required for this study.

## Results

In total, 209,755 regular sleeping pill users (i.e., use for >3 months) were enrolled between 2000 and 2013. CKD was observed in 18,131 of 186,654 sleeping pill users and in 22,154 of 373,308 nonusers. Figure 1 shows the research design of this study.

Table 2 shows the comorbidities and demographic features of the sleeping pill users (186,654) and nonusers (373,308) from 2000 to 2013. People >60 years of age comprised 40% of both cohorts with similar age and sex distributions. Men accounted for 58% of the population in both groups. Sleeping pill use occasionally occurred concurrently in the patients with comorbidities such as hypertension, diabetes mellitus, stroke, LC, COPDa, CHF, and CAD (all *p* < 0.05), with a similar prevalence (*p* = 0.485). No cooccurrence was observed in all the patients with hyperlipidemia. We also observed a higher prevalence of sleeping pill use in northern Taiwan, areas of higher urbanization, and our local hospital.

**TABLE 2:** Demographic characteristics and comorbidities in cohorts with and without sleeping pills use.

Variables	Total	Sleeping pills use		*p* value
		Yes	No	
N (%)	559,962	186,654 (33.33)	373,308 (66.67)	
Age, year				0.999
18–29	72,924 (13.02)	24,308 (13.02)	48,616 (13.02)	
30–39	79,017 (14.11)	26,339 (14.11)	52,678 (14.11)	
40–49	95,658 (17.08)	31,886 (17.08)	63,772 (17.08)	
50–59	91,041 (16.26)	30,347 (16.26)	60,694 (16.26)	
≧60	221,322 (39.52)	73,774 (39.52)	147,548 (39.52)	
Sex				0.999
** **Female	233,547 (41.71)	77,849 (41.71)	155,698 (41.71)	
** **Male	326,415 (58.29)	108,805 (58.29)	217,610 (58.29)	
Comorbidity				
Hypertension	79,325 (14.17)	33,112 (17.74)	46,213 (12.38)	<0.001
DM	59,735 (10.67)	21,194 (11.35)	38,541 (10.32)	<0.001
CHF	11,073 (1.98)	4,112 (2.20)	6,961 (1.86)	<0.001
Stroke	39,517 (7.06)	14,534 (7.79)	24,983 (6.69)	<0.001
COPDa	39,346 (7.03)	15,624 (8.37)	23,722 (6.35)	<0.001
LC	29,967 (5.35)	8,957 (4.80)	21,010 (5.63)	<0.001
CAD	38,602 (6.89)	12,616 (6.76)	25,986 (6.96)	<0.001
Hyperlipidemia	16,327 (2.92)	5,314 (2.85)	11,013 (2.95)	0.485
CCI_R	0.49 ± 1.88	0.76 ± 2.52	0.36 ± 1.44	<0.001
Season				<0.001
Spring (March–May)	148,649 (26.55)	48,130 (25.79)	100,519 (26.93)	
Summer (June–August)	139,393 (24.95)	47,206 (25.29)	92,487 (24.77)	
Autumn (September–November)	130,474 (23.30)	46,851 (25.10)	83,623 (22.40)	
Winter (December–February)	141,146 (25.21)	44,467 (23.82)	96,679 (25.90)	
Location				<0.001
Northern Taiwan	209,137 (37.35)	60,208 (32.26)	148,929 (39.89)	
Middle Taiwan	163,183 (29.14)	62,875 (33.69)	100,308 (26.87)	
Southern Taiwan	147,442 (26.33)	48,121 (25.78)	99,321 (26.61)	
Eastern Taiwan	36.888 (6.59)	14,341 (7.68)	22,547 (6.04)	
Outlets Islands	3,312 (0.59)	1,109 (0.59)	2,203 (0.59)	<0.001
Urbanization level				0.701
1 (the highest)	180,200 (32.18)	51,949 (27.83)	128,251 (34.36)	<0.001
2	231,075 (41.27)	76,190 (40.84)	154,885 (41.49)	<0.001
3	49,432 (8.83)	19,899 (10.66)	29,533 (7.91)	<0.001
4 (the lowest)	99,255 (17.73)	38,616 (20.69)	60,639 (16.24)	<0.001
Level of care				<0.001
Medical center	159,653 (28.51)	50,524 (27.07)	109,129 (29.23)	
Region hospital	186,615 (33.33)	78,429 (42.02)	108,186 (28.98)	
Local hospital	213,694 (38.16)	57,701 (30.91)	155,993 (41.79)	
Insured premium				<0.001
<18,000	483,656 (86.37)	161,245 (86.26)	327,095 (87.62)	
18,000–34,999	54,313 (9.70)	18,010 (9.65)	36,303 (9.72)	
≧35,000	21,993 (3.93)	7,399 (3.96)	14,594 (3.91)	
Medications				
NSAIDs	110,876 (19.80)	44,124 (23.64)	66,752 (17.88)	<0.001
ACEI and ARB	57,034 (10.19)	23,245 (12.45)	33,789 (9.05)	<0.001
Antibacterial agents	12,603 (2.25)	4,592 (2.46)	8,011 (2.15)	<0.001
Antifungal agents	9,476 (1.69)	3,352 (1.80)	6,124 (1.64)	<0.001
Antiviral agents	18,714 (3.34)	6,063 (3.25)	12,651 (3.39)	0.006
Immunosuppressive agents	8,635 (1.54)	3,034 (1.63)	5,601 (1.50)	<0.001

Chi-square/Fisher’s exact test; continuous variable: *t*-test. *p value <0.05. CKD, chronic kidney disease; DM, diabetes mellitus; CHF, congestive heart failure; COPDa, chronic obstructive pulmonary disease and allied conditions; LC, chronic liver disease and cirrhosis; CAD, coronary artery disease; CCI_R, Charlson comorbidity index with DM, CHF, stroke, COPDa, LC, CAD, and hyperlipidemia removed. NSAIDs, nonsteroidal anti-inflammatory drugs; ACEI, angiotensin-converting enzyme inhibitors; ARB, angiotensin receptor blocker.

The incidence rate of progression to CKD was 978 per 100,000 person-years for the sleeping pill users compared with 576 per 100,000 for the nonusers. The incidence rate of CKD was 1.69-fold higher in those who used sleeping pills than in the nonusers. We further evaluated the risk of ESRDd after CKD diagnosis in those who continuously used sleeping pills using the Cox regression model, which revealed an increased risk of CKD progression (adjusted HR [aHR] = 7.542; 95% CI: 4.267–10.156; *p* < 0.001; Table 3).

**TABLE 3:** Factors of ESRD among CKD patients by using Cox regression.

Variables^a^	Population	Events	PYs	Rate	Crude HR	95% CI	*p* value	Adjusted HR	95% CI	*p* value
Sleeping pills^a^	18,131	7,862	412,986.55	1,903.69	7.688	4.832–10.996	<0.001*	7.542	4.267–10.156	<0.001*

PYs, person-years; rate, per 10^5^ PYs; HR, hazard ratio; CI, confidence interval; adjusted HR: adjusted for all the variables of age, gender, and comorbidities, including HTN, hypertension; DM, diabetes; CHF, congestive heart failure; stroke; COPDa, chronic obstructive pulmonary disease and allied conditions; LC, chronic liver disease; cirrhosis; CAD, coronary artery disease; and hyperlipidemia. *p value <0.05.

Sleeping pill users were shown to have an increased risk of CKD compared with the nonusers after univariate and multivariate analyses were performed with adjustments for age, sex, and comorbidities (aHR = 1.806; 95% CI: 1.617–2.105; *p* < 0.001).

The aHR for CKD was much greater for elderly individuals (age: 60 years; aHR = 4.678: 95% CI: 4.351–5.209; *p* < 0.001) than for those aged 18–29 years. Patients with comorbidities such as hypertension, diabetes mellitus, stroke, LC, COPDa, CHF, CAD, and hyperlipidemia exhibited an increased risk for developing CKD. Higher insurance premiums, lower urbanization, and central facilities were all associated with an increased risk of CKD. Medications such as NSAIDs, ACEI, and ARB were all associated with an increased risk of CKD. Furthermore, each increase in CCI score led to a 1.025% increase in CKD risk (table not shown). After stratification by sleeping pill use, a risk increase was apparent according to sex (both), age, and comorbidities such as hypertension, diabetes mellitus, CHF, stroke, COPDa, LC, CAD, and nephrotoxic agents. Hyperlipidemia (aHR = 1.046; 95% CI: 0.937–1.222; *p* = 0.108) and higher insurance premium of 35,000 Taiwanese dollars (aHR = 0.852; 95% CI: 0.673–1.207; *p* = 0.246) were not significant contributors to the risk after stratification (Table 1).

**FIGURE 2:**
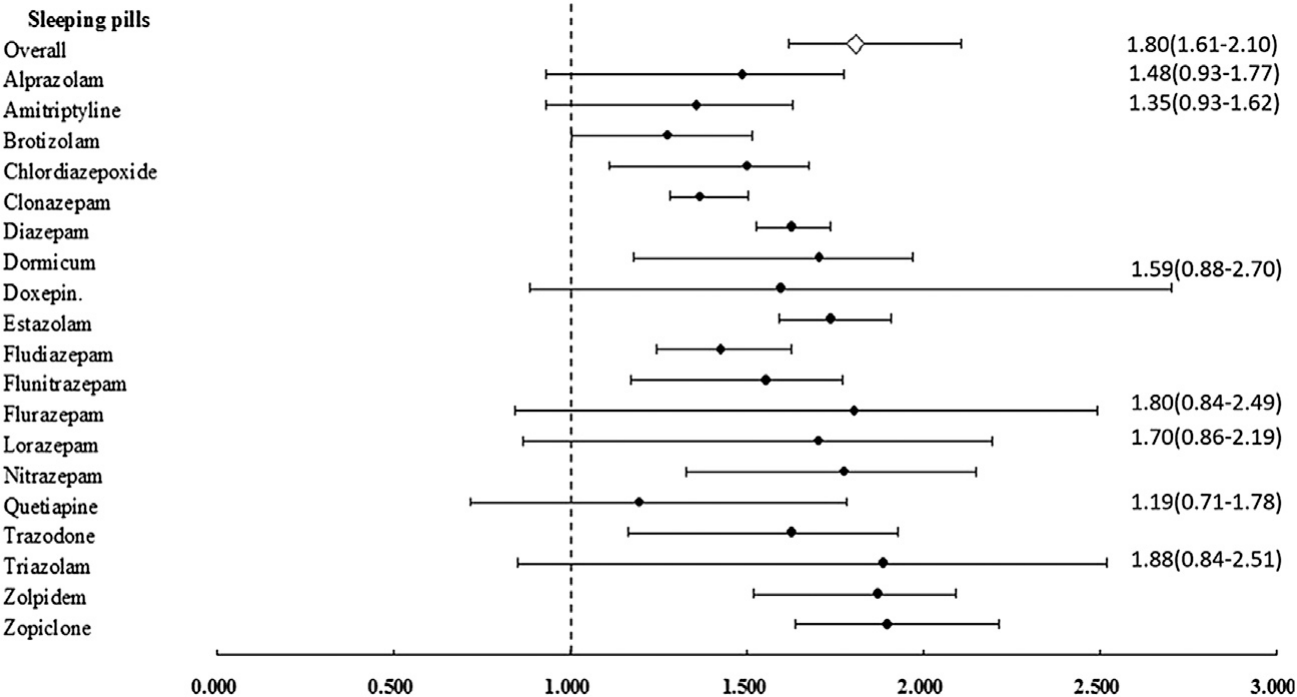
Adjusted hazard ratio of chronic kidney disease (CKD) in individuals with affected sleeping pills. Error bars indicate 95% confidence interval (CI).

**FIGURE 3:**
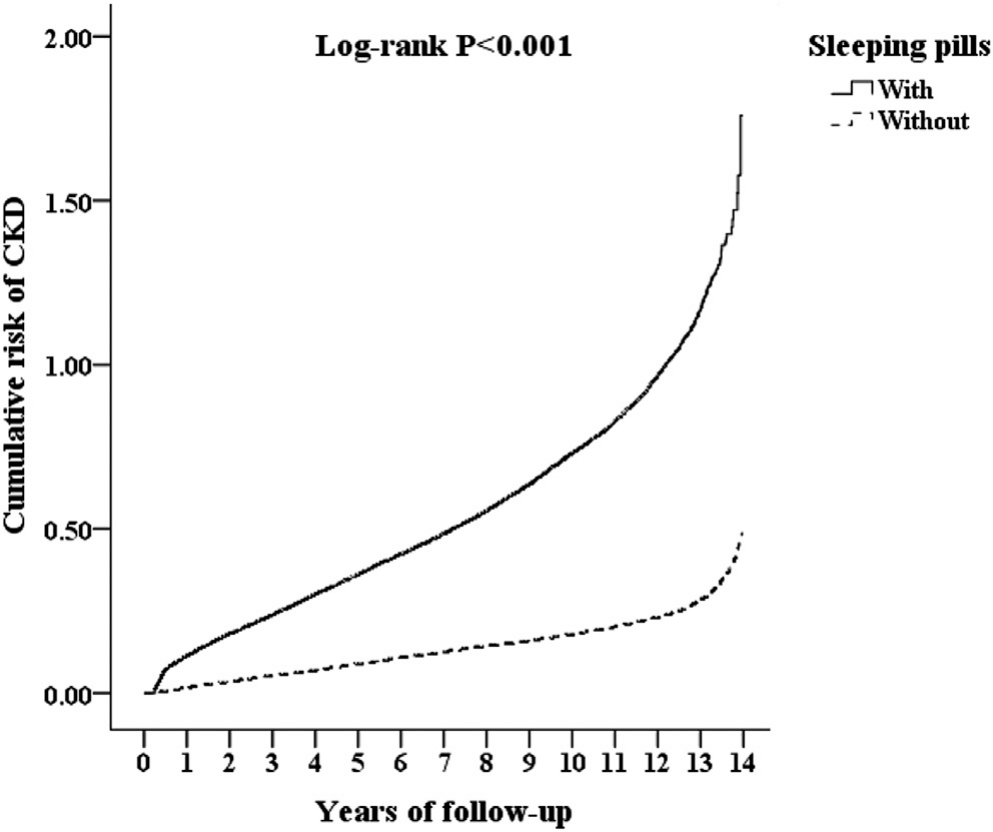
Kaplan–Meier analysis for cumulative risk of CKD among those aged 18 and over, stratified by sleeping pills with the log-rank test.

After stratification of the sleeping pills by generic drug classification, most sleeping pills were associated with an increased risk of CKD, except for alprazolam (aHR = 1.487; 95% CI: 0.931–1.772; *p* = 0.068), amitriptyline (aHR = 1.358; 95% CI: 0.930–1.628; *p* = 0.070), doxepin (aHR = 1.595; 95% CI: 0.885–2.702; *p* = 0.129), flurazepam (aHR = 1.804; 95% CI: 0.841–2.490; *p* = 0.158), lorazepam (aHR = 1.702; 95% CI: 0.866–2.192; *p* = 0.137), quetiapine (aHR = 1.196; 95% CI: 0.715–1.783; *p* = 0.286), and triazolam (aHR = 1.886; 95% CI: 0.848–2.519; *p* = 0.153) (Table 4 and Figure 2). Compared with the nonusers, the sleeping pill users demonstrated a significantly higher cumulative risk of CKD based on the Kaplan–Meier analyses (*p* < 0.001); (Figure 3).

**TABLE 4:** Crude and adjusted odds ratios of CKD associated with various sleeping pills administration during the follow-up period in the study cohort.

Variables^a^	Crude HR	95% CI	*p* value	Adjusted HR	95% CI	*p* value
Sleeping pills^a^	1.818	1.789–1.902	<0.001	1.806	1.617–2.105	<0.001*
Alprazolam	1.594	1.030–1.895	0.018	1.487	0.931–1.772	0.068
Amitriptyline	1.498	1.002–1.706	0.048	1.358	0.930–1.628	0.070
Brotizolam	1.398	1.111–1.564	0.003	1.276	1.003–1.513	0.046*
Chlordiazepoxide	1.506	1.2244–1.765	<0.001	1.501	1.110–1.673	<0.001*
Clonazepam	1.449	1.398–1.501	<0.001	1.368	1.282–1.501	<0.001*
Diazepam	1.799	1.642–1.836	<0.001	1.627	1.527–1.736	<0.001*
Dormicum	1.813	1.299–2.012	0.001	1.705	1.179–1.970	<0.001*
Doxepin	1.701	0.972–2.982	0.134	1.595	0.885–2.702	0.129
Estazolam	1.906	1.807–2.072	<0.001	1.736	1.591–1.907	<0.001*
Fludiazepam	1.535	1.384–1.803	0.007	1.426	1.244–1.624	<0.001*
Flunitrazepam	1.678	1.271–1.895	<0.001	1.555	1.172–1.770	<0.001*
Flurazepam	2.174	0.973–2.997	0.070	1.804	0.841–2.490	0.158
Lorazepam	1.911	1.012–2.551	0.038	1.702	0.866–2.192	0.137
Nitrazepam	1.924	1.568–2.497	<0.001	1.775	1.328–2.147	<0.001*
Quetiapine	1.355	0.864–2.012	0.246	1.196	0.715–1.783	0.286
Trazodone	1.801	1.245–2.131	0.001	1.627	1.162–1.927	<0.001*
Triazolam	1.996	0.931–2.724	0.070	1.886	0.848–2.519	0.153
Zolpidem	2.019	1.672–2.486	<0.001	1.872	1.519–2.091	<0.001*
Zopiclone	1.905	1.539–2.11	<0.001	1.897	1.638–2.213	<0.001*

HR, hazard ratio; CI, confidence interval; adjusted HR, adjusted for all the variables of age, gender, and comorbidities, including HTN, hypertension; DM, diabetes; CHF, congestive heart failure; stroke; COPDa, chronic obstructive pulmonary disease and allied conditions; LC, chronic liver disease and cirrhosis; CAD, coronary artery disease; hyperlipidemia. *p value <0.05.

^a^ Without the disease or medication as reference.

## Discussion

After adjusting for potential confounders, sleeping pill use was correlated with a significant (80%) increase in the risk of CKD (Table 1). In addition, this study revealed that all the sleeping pills except alprazolam, amitriptyline, doxepin, flurazepam, lorazepam, quetiapine, and triazolam were associated with an increased risk of CKD. No study has previously rigorously examined the adverse effects of sleeping pill use (including benzodiazepines). The prevalence of sleeping pill use in a previous Brazilian population study was 7.6% and was closely associated with female sex, age ≥60 years, and smoking (Kodaira and Silva, 2017).

A significantly higher prevalence of sleeping pill use (5.5%) was observed among morbidly obese (body mass index (BMI) ≥ 35 kg/m^2^) men and underweight (BMI < 18.5 kg/m^2^) women in a Canadian study (Vozoris and Leung, 2011). In this study, the prevalence of sleeping pill use generally increased with age. A previous study reported that 2–5% of young adults used benzodiazepines or nonbenzodiazepine omega-receptor agonists and the use was particularly high in older adults, women, Caucasians, and current smokers. In the study by Winkelmayer et al., hypertension and diabetes mellitus were not associated with benzodiazepine use, while several other comorbid conditions such as CAD, CHF, peripheral vascular disease, COPD, and malignancy were significantly associated with the use of benzodiazepines (Winkelmayer et al., 2007). These comorbidity results are compatible with the findings in our study regarding CAD, CHF, and COPDa. Conversely, our study demonstrated that sleeping pill use was more frequently observed in patients with hypertension, diabetes mellitus, stroke, liver cirrhosis, and hyperlipidemia. The percentages of patients with CKD and ESRD using sleeping pills are seemingly rising, owing to the significant psychiatric burden of physical illness (Pham et al., 2017).

According to Winkelmayer et al., dialysis patients using benzodiazepines were predominantly Caucasian (77% vs. 60%, *p* = 0.001) and female (53% vs. 45%, *p* = 0.002), had lung disease (odds ratio [OR] = 1.43; 95% CI: 1.03–1.98), and seldom had cerebrovascular diseases (Winkelmayer et al., 2007). Benzodiazepines such as temazepam, lorazepam, alprazolam, and clonazepam have reportedly been prescribed in dialysis populations for the treatment of anxiety, sleep disorders, restless leg syndrome, and depression (Fukuhara et al., 2006). Sedative use was previously associated with increased mortality; however, the reasons were unknown (Hausken et al., 2007). Although our study confirmed an association between sleeping pill use and CKD risk, research discussing this relationship is limited. Mechanisms underlying the association between sleeping pill use and the deterioration of kidney function are still unclear. According to our study, those using sleeping pills usually had underlying comorbidities (e.g., concomitant hepatic disease or CHF), which may contribute to the risk of nephrotoxicity in patients with advancing age, volume depletion, and selected high-risk therapies such as NSAIDs, aminoglycosides, ACE inhibitors, and radiographic contrast media (Ponticelli et al., 2015). In relation to the previous aspect, it should be taken into account that when a patient uses a high number of drugs concomitantly, generally, it is considered polypharmacy from 5. This is an important aspect when the outcome is renal damage. Benzodiazepines are the most common class drugs used as sleep aids. Their action is based on an inhibitory neurotransmitter called GABA, which specifically acts in a transmembrane structure on GABA receptors (GABA agonists) known as the GABAA receptor protein complex. This results in the opening of chloride channels with the consequent influx of ions into neurons, causing hyperpolarization. Undesirable side-effects are due to the prolonged action caused by renal or liver failure, as the liver and kidney are the primary organs for benzodiazepine metabolism and excretion. Benzodiazepines that include active metabolites such as clorazepate, chlordiazepoxide, diazepam, and flurazepam should be prohibited in patients with renal insufficiency and those with ESRD (Wyne et al., 2010). Renal and nonrenal drug clearance mechanisms, such as the CYP3A4 clearance of benzodiazepines, may be compromised in CKD and ESRD conditions, leading to drug side-effects and possible cumulative effects on the kidneys (Velenosi et al., 2012; Thomson et al., 2015). Both the dibenzodiazepine derivative quetiapine (an atypical or second-generation antipsychotic) and olanzapine have been reported to induce chronic interstitial nephritis (He et al., 2013). Furthermore, propylene glycol is contained in parenteral formulations of benzodiazepines (e.g., lorazepam, chlordiazepoxide, and diazepam), which may cause acute kidney injury, proximal tubule injury, hyperosmolarity, and sepsis-like syndrome (Cawley, 2001). Worsening renal failure and metabolic acidosis have both been correlated with prolonged infusion of solutions containing propylene glycol. Withholding these agents has been recommended in critically ill patients with a creatinine clearance of ≤30 ml/min (Cawley, 2001). Some benzodiazepines and antidepressants/mood stabilizers, such as amitriptyline and doxepin, have been correlated with rhabdomyolysis (Coco and Klasner, 2004).

However, in our study, the use of alprazolam, amitriptyline, doxepin, flurazepam, lorazepam, quetiapine, or triazolam was not significantly associated with CKD. It seems that the lack of statistical significance for these drugs is due to a problem of imprecision. It is not appropriate to state that these drugs are risk-free. According to the studies of Kim et al. and Choi et al., CKD was strongly suggested to be significantly associated with long sleep duration (≥9 h/day). However, these studies failed to report on the effect of benzodiazepine use (Choi et al., 2017; Kim et al., 2017). We propose that benzodiazepine overuse may simultaneously prolong sleep duration and lead to CKD. In our study, hyperlipidemia and higher insurance premium were not associated with CKD risk associated with sleeping pill use. Associations between hyperlipidemia and sleeping pill use are therefore still not clear. The collinearity of hypertension and hyperlipidemia may explain this phenomenon. In addition, the use of lipid-lowering drugs may have renal protective effects that alleviate the renal toxicity associated with sleeping pill use (McWilliam et al., 2018).

Individuals with low insurance premiums are often equal to the group with a low socioeconomic status in Taiwan who were at risk of CKD events in our study. A study by Bello et al. previously reported that people of lower socioeconomic status have a higher risk of ischemic heart disease events than those of higher socioeconomic status (Bello et al., 2008). In adults with CKD, death risk can be attributed to nonrestorative sleep, short sleep duration, and restless leg syndrome (Ricardo et al., 2017). Thus, a balance between adequate sleep quality and evaluating the impact of sleeping pills in this population remains to be determined. The strengths and limitations of our study may be similar to those in most retrospective studies. The greatest strengths of this study were its long follow-up duration of 13 years and large-scale population-based data source. Patients who died within 90 days after the index date were excluded to reduce survival bias. To reduce off-label use of sleeping pills, patients with obstructive sleep apnea and nonapnea sleep disorders were excluded from our study. Our study had some pertinent limitations. First, data from insurance claims did not include measurements of microalbuminuria and serum creatinine; therefore, we may not have included patients with early CKD in our study. The frequency of sleeping pill use may have been increasing owing to the mental burden of CKD and ESRD. Although we excluded patients who had CKD and ESRD before entering our study, analysis of antecedents and consequences may have been confused or misinterpreted. Second, the results of our study are inappropriate for interpretation in patients with CKD of different ethnicities, as our study cohort was composed mostly of Taiwanese people. Third, we failed to adjust several possible unmeasured confounding variables for CKD that were unavailable in the NHIRD dataset (e.g., individual blood pressure control status, diet preference, and smoking status), although we adjusted for potential confounders during the statistical analysis. Finally, the study method defined sleeping pills use after 2000, which could result in underestimation of the drug effects on CKD if the drug was administered before 2000.

## Conclusion

In brief, sleeping pill use and the risk of CKD significantly were correlated (101%). The risk and impact on public health should be reevaluated because sleeping pills are a commonly prescribed medication. However, the use of benzodiazepines such as alprazolam, flurazepam, lorazepam, and triazolam, and other agents such as amitriptyline, doxepin, and quetiapine may be warranted in susceptible CKD cases. Additional studies, particularly prospective randomized trials with longer prescription durations, are required to confirm our findings.

## Data Availability Statement

The original contributions presented in the study are included in the article/Supplementary Material; further inquiries can be directed to the corresponding authors.

## Ethics Statement

This study was approved by the Institutional Review Board of the Tri-Service General Hospital (study approval code: TSGHIRB No. 2-106-05-029). Patient consent was not required for this study as the NHIRD had encrypted patient personal information and provided researchers with anonymous identification numbers associated with relevant claim information, including sex, date of birth, medical services received, and prescriptions.

## Author Contributions

C-YL contributed to manuscript writing. C-HC contributed to data collection and analysis. K-CL contributed to data interpretation. C-YC contributed to data interpretation. S-SY contributed to data interpretation. W-CC contributed to data collection and analysis. C-CW contributed to the idea of the manuscript and manuscript editing.

## Funding

This study was supported by research grants from the Tri-Service General Hospital (TSGH-C05-110033 and TSGH-B-110012) and the Ministry of Science and Technology (MOST107-2314-B-016-004-MY3), Taiwan. The funders had no role in study design, data collection and analysis, decision to publish, or preparation of the manuscript. The work was supported by the School of Public Health, National Defense Medical Center, Taipei, Taiwan.

## Conflict of Interest

The authors declare that the research was conducted in the absence of any commercial or financial relationships that could be construed as a potential conflict of interest.
